# Massive Foramen Magnum Meningioma Mimicking a Stroke

**DOI:** 10.7759/cureus.84525

**Published:** 2025-05-21

**Authors:** Alexis D Navarro, Herminigildo H Gan

**Affiliations:** 1 Neurology, Jose R. Reyes Memorial Medical Center, Manila City, PHL

**Keywords:** c1 laminectomy, foramen magnum lesion, meningothelial meningoma, skull base meningioma, stroke mimic

## Abstract

Foramen magnum meningiomas (FMMs) are skull base tumors that arise from the arachnoid layer of the meninges at the cranio-cervical junction. They typically present gradually, with symptoms ranging from asymmetric, progressive quadriparesis and sensory impairment to involvement of the lower cranial nerves. We report a case of a 50-year-old Asian female who presented with sudden-onset left-sided numbness. Neurologic examination revealed hemisensory loss over her left extremities. Imaging studies showed a meningioma within the spinal canal at the level of C1-C2. She underwent surgical intervention, and there was resolution of the symptoms upon discharge.

This case highlights the importance of recognizing atypical manifestations of FMM, such as a sudden-onset hemianesthesia, mimicking a stroke.

## Introduction

Meningiomas are benign, slow-growing tumors that originate from arachnoid cells, particularly meningothelial cells, which form part of the meninges surrounding the brain and spinal cord [1]. They constitute a significant portion of all primary intracranial neoplasms - around 14.3% to 19% [2]. However, only 1.8% to 3.2% of meningiomas form near the foramen magnum, referred to as foramen magnum meningiomas (FMMs). Their indolent growth at the cranio-cervical junction, where the brain meets the spinal cord, makes the diagnosis complex, and the time between the onset of symptoms and diagnosis varies [2,3]. Common manifestations arise from the compression of the brainstem and spinal cord, which causes chronically progressive symptoms like gait disturbances, dysphagia, and various neurological deficits that may lead to delays in diagnosing and make treatment more difficult [4]. When massive, it may also manifest symptoms that indicate an increased intracranial pressure [5]. We report a case of FMM presenting with sudden-onset hemianesthesia that resembles a stroke.

## Case presentation

A 50-year-old Asian female presented at our clinic with sudden-onset left-sided numbness. She has been a known hypertensive for three years, maintained on Losartan 50 mg tablet once daily, with good compliance, but has no blood pressure (BP) monitoring. Upon general physical examination, vital signs were within normal limits. On neurologic examination, she was awake, conversant, and oriented to three spheres and was able to follow commands. She had no cranial nerve deficits, normal motor strength at 5/5 on manual muscle testing (MMT), and hemisensory loss over her left extremities at 50%. Initial blood counts, coagulation parameters (Table 1), and blood chemistry tests (Table 2) were normal.

**Table 1:** Complete blood count (CBC) and coagulation profile (PT, aPTT) showing normal results PT: Prothrombin Time; INR: International Normalized Ratio; aPTT: Activated Partial Thromboplastin Time

Hematology			
COMPLETE BLOOD COUNT	RESULT	UNIT	NORMAL RANGE
Hemoglobin	140	g/L	120-160
Hematocrit	0.45	-	0.37-0.47
Red Blood Cell	5.51	X10^12^/uL	4.0-5.2
White Blood Cell	7.2	X10^9^/L	5.0-11.0
Neutrophil	61	%	35-66
Lymphocyte	30.4	%	24-44
Basophil	0.7	%	0-2
Monocyte	4.7	%	03-Jun
Eosinophil	2.3	%	01-Mar
Platelet	359	X10^9^/uL	150-400
COAGULATION	RESULT	UNIT	NORMAL RANGE
PT	12.8	seconds	11.7-15.3
INR	0.97	-	0.80-1.20
%	100	^%^	70-100
PTT	24.9	seconds	24.8-34.4

**Table 2:** Blood chemistry panel showing normal results LDL: Low Density Lipoprotein; HDL: High Density Lipoprotein

Blood Chemistry			
	RESULT	UNIT	NORMAL RANGE
Sodium	143.1	mmol/L	137.0-145.0
Potassium	3.9	mmol/L	3.5-5.1
Creatinine	64.3	umol/L	62.0-106.0
Fasting Blood Sugar	4.9	mmol/L	4.1-5.9
Total Cholesterol	5.1	mmol/L	<5.2
LDL	2.4	mmol/L	<2.6
HDL	1.8	mmol/L	>1.0
Triglycerides	1.6	mmol/L	<1.7

**Figure 1:**
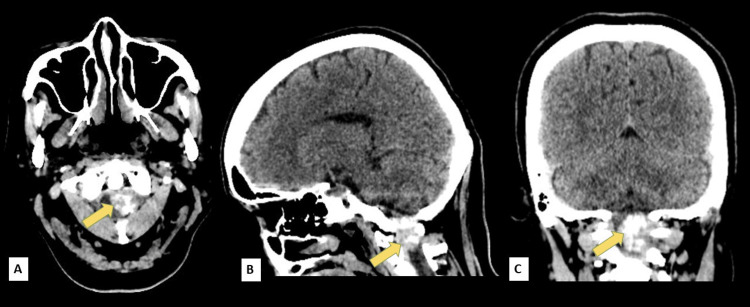
Plain cranial computed tomography (CT) scan in (A) axial, (B) sagittal, and (B) coronal views showing a well-defined extra-axial hyperdense mass with intralesional calcifications measuring 3.2x2.0x1.9 cm (CCxWxAP) within the spinal canal at the level of C1-C2 (arrow) CC: Craniocaudal; W: Width; AP: Anteroposterior

**Figure 2:**
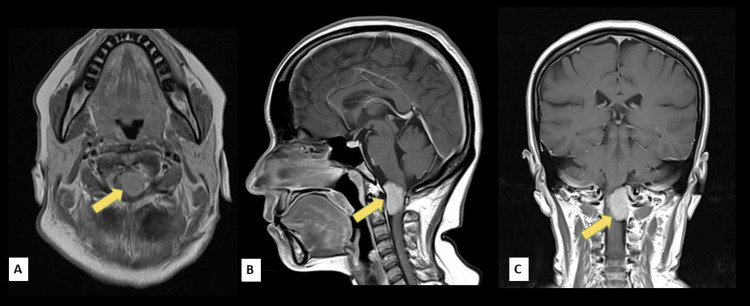
T1-weighted, post-gadolinium cranial magnetic resonance (MR) imaging in (A) axial, (B) sagittal, and (C) coronal views showing a well circumscribed, contrast-enhancing extra-axial mass lesion measuring approximately 2.9x2.1x3.1 cm (CCxWxAP) within the spinal canal at the level of C1-C2, extending from the inferior clivus to the level of the C2 vertebra (arrow) CC: Craniocaudal; W: Width; AP: Anteroposterior

Our initial impression during this time was a lacunar syndrome, pure sensory, probably secondary to small vessel disease. The cranial computed tomography (CT) scan and subsequent cranial magnetic resonance (MR) imaging revealed no evidence of acute infarction but with an incidental finding of meningioma within the spinal canal at the level of C1-C2 (Figures 1, 2). 

Surgical intervention was then planned, and the patient’s informed consent was obtained. The procedure was done by a consultant neurosurgeon with 14 years of experience. She underwent surgery using the left far lateral approach. The patient was placed in the park-bench position. A hockey-stick incision was made extending through the deep layers and to the level of the suboccipital bone. C1 laminectomy was performed to allow for a wider dural opening and adequate lesional exposure. The dura was opened in a Y-shaped fashion with a more inferior extension for more lateral mobilization of the dural flaps. The tumor was noted to be encapsulated and firmly attached to the dura. The tumor was devascularized and centrally debulked. The vertebral arteries were dissected free. Once the tumor was centrally debulked, its capsule was mobilized, and all of the pial adhesions were sharply divided, and small tumor feeders were coagulated. Complete excision was done with coagulation of the dural base, and the tumor was then resected. The surgery proceeded without complications, and the patient remained hemodynamically stable throughout the procedure. Histopathologic examination of the tumor showed epithelioid cells with round to oval nuclei, inconspicuous nucleoli, lightly eosinophilic cytoplasm, and indistinct cytoplasmic borders, arranged in a syncytial configuration. Also seen are numerous randomly dispersed, calcific concretions. These findings were then consistent with a meningothelial meningioma, WHO grade I. She was started on dexamethasone 4 mg/IV every 8 hours, on a tapering dose, and gradually, there was resolution of sensory loss in her left extremities. She was then discharged after she improved 11 days after surgery. On follow-up, she had no recurrence of her previous symptoms, and there was no occurrence of new-onset deficits.

## Discussion

FMMs are slow-growing tumors arising from the arachnoid cells at the dura mater of the cranio-cervical junction. They account for approximately 1.8 to 3.2% of intracranial meningiomas [2,3] and are prevalent among adults over the age of 60, with a greater occurrence in females [6].

The slow growth of foramen magnum meningiomas accounts for the longer latency between the start of symptoms and diagnosis, resulting in an insidious clinical presentation [2]. FMM may present with a progressive spastic quadriparesis (or hemiparesis) because of the compression of the pyramidal tracts at the cervico-medullary junction, where the lower extremities are usually affected before the arms because of the somatotopic organization of the corticospinal tract. There may also be a deep and aching pain in the neck and occipital region, often radiating down the upper cervical spine or shoulder. Furthermore, lower cranial nerves may also be involved, leading to dysphagia, dysarthria, tongue atrophy or fasciculations, and weakness of the sternocleidomastoid or trapezius. Sensory changes may also be experienced, characterized as ipsilateral loss of proprioception, fine touch, vibration, and contralateral loss of pain and temperature [7].

In our patient, instead of a gradual development of symptoms, she presented with sudden-onset left-sided numbness, which could be due to an acute compression of the ipsilateral dorsal column of the spinal cord. Moreover, in contrast to the typical somatotopic pattern of involvement of the lower extremity followed by the upper extremity, our patient presented with the simultaneous involvement of both upper and lower extremities. The former could be explained through the concept of neural adaptation, which is a phenomenon of neuronal activity declining in response to sustained or repeated stimulation. Neural adaptation may help conceal symptoms in the case of a tumor such as FMM. FMMs and other tumors usually grow slowly. To preserve function, the brain may reorganize neural networks, causing early symptoms to be concealed. Furthermore, a high-pass filter is introduced by neural adaptation, which highlights environmental fluctuations as opposed to continuous stimuli. As a result, the nervous system becomes less sensitive to the tumor's continuous pressure. Eventually, the compensatory systems may become exhausted as the tumor grows, which could cause symptoms to worsen more quickly. When these processes are depleted, the clinical course may appear. Just like in our case, despite the massive size of the FMM, our patient only experienced sudden-onset left-sided numbness [8,9].

FMMs can be difficult to diagnose because of their variable clinical presentations and slow growth. Even with MR imaging, the average time between symptoms and diagnosis is about 30.8 months. Multiple sclerosis, amyotrophic lateral sclerosis, syringomyelia, cervical spondylosis, and stroke are among the differential diagnoses to take into account. High-resolution pictures of soft-tissue anatomy are provided by MR imaging, the recommended technique for detecting malignancies in the foramen magnum. Computed tomography (CT) scans can also be used to evaluate the surgical corridor and osseous structure. Furthermore, CT or MR angiography can be used to visualize how the tumor relates to vascular structures like the vertebral artery [9].

Tumor excision is the primary treatment for foramen magnum meningiomas. The surgical approach is determined by the tumor's anatomical position, in which tumors located posterior or postero-lateral to the spinal cord are operated through a posterior suboccipital craniotomy, providing direct access with minimal risk to anterior neurovascular structures. On the other hand, anterior or antero-lateral tumors require a far-lateral or transcondylar route, for safe dissection and to avoid injury to the medulla, spinal cord, or cranial nerves. The extent of tumor resection significantly influences prognosis. Complete excision is associated with reduced recurrence and improved neurological outcomes. However, when tumors encase critical structures, subtotal resection may be necessary to preserve neurological function. When surgery is contraindicated due to patient comorbidities, adjunctive radiotherapy may be employed to limit tumor progression. The management approach should be individualized based on the tumor’s anatomical features and the patient’s neurological condition, aiming to achieve the most complete resection possible while prioritizing the preservation of neural function [10].

## Conclusions

This case emphasizes an atypical presentation of a foramen magnum meningioma, manifesting as sudden-onset hemianesthesia mimicking a stroke. Although these tumors usually present gradually and with progressive neurological deficits, they may present solely with an acute sensory deficit despite being massive. Clinicians should maintain a broad differential when evaluating sudden focal deficits, as rare structural lesions like FMM may mimic more common conditions such as stroke. Early recognition through appropriate imaging is essential for timely management and improved outcomes. Treatment should also be tailored to the tumor’s location and the patient’s condition, aiming to safely remove as much of the tumor as possible while protecting brain and nerve function.
